# DMET™ (Drug Metabolism Enzymes and Transporters): a pharmacogenomic platform for precision medicine

**DOI:** 10.18632/oncotarget.9927

**Published:** 2016-06-09

**Authors:** Mariamena Arbitrio, Maria Teresa Di Martino, Francesca Scionti, Giuseppe Agapito, Pietro Hiram Guzzi, Mario Cannataro, Pierfrancesco Tassone, Pierosandro Tagliaferri

**Affiliations:** ^1^ ISN-CNR, Roccelletta di Borgia, Catanzaro, Italy; ^2^ Department of Experimental and Clinical Medicine, Medical Oncology Unit, Mater Domini Hospital, Magna Graecia University, Salvatore Venuta University Campus, Catanzaro, Italy; ^3^ Department of Medical and Surgical Sciences, Magna Graecia University, Catanzaro, Italy; ^4^ ICAR-CNR, Cosenza, Italy

**Keywords:** pharmacogenomics, single nucleotide polymorphism, DMET™, ADME genes, biomarkers

## Abstract

In the era of personalized medicine, high-throughput technologies have allowed the investigation of genetic variations underlying the inter-individual variability in drug pharmacokinetics/pharmacodynamics. Several studies have recently moved from a candidate gene-based pharmacogenetic approach to genome-wide pharmacogenomic analyses to identify biomarkers for selection of patient-tailored therapies. In this aim, the identification of genetic variants affecting the individual drug metabolism is relevant for the definition of more active and less toxic treatments. This review focuses on the potentiality, reliability and limitations of the DMET™ (Drug Metabolism Enzymes and Transporters) Plus as pharmacogenomic drug metabolism multi-gene panel platform for selecting biomarkers in the final aim to optimize drugs use and characterize the individual genetic background.

## INTRODUCTION

**Figure 1:**
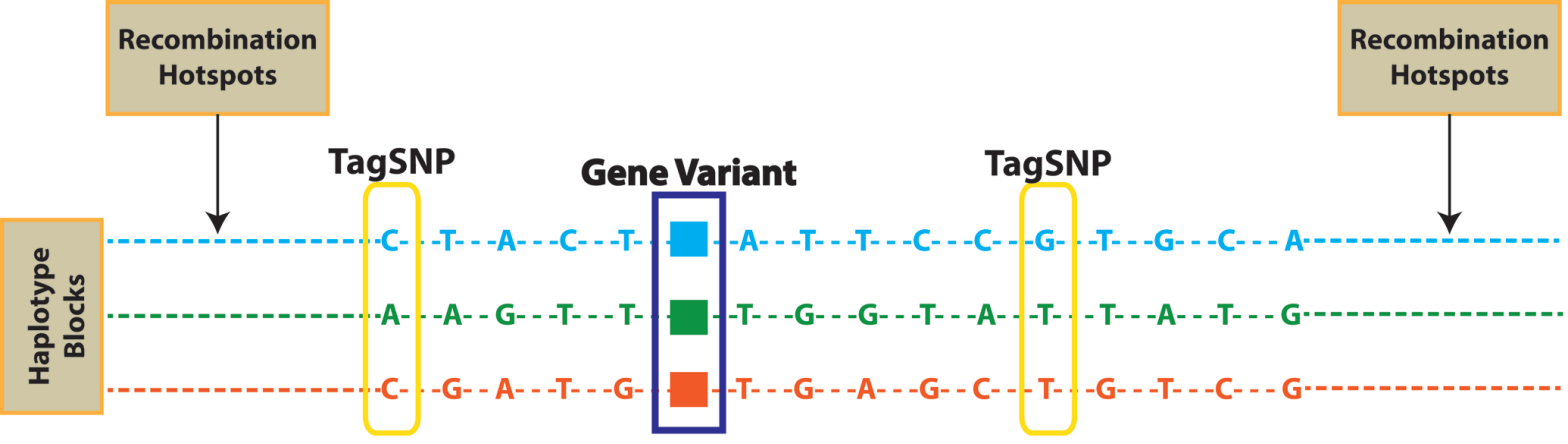
TagSNPs and recombination hotspots Single nucleotide polymorphisms (SNPs) in linkage disequilibrium (LD) are coheredited in haplotype blocks. TagSNPs are used to identify gene variants potentially correlated to phenotypes, withouth the need to genotpype all SNPs included in each haplotype block.

The Human Genome [1, 2] and the International HapMap projects [3, 4] have provided a great opportunity for the understanding of the complex genomic architecture of disease susceptibility and inter-individual drug response variability. In clinical practice, the knowledge of genetic factors influencing drug efficacy and safety is of major relevance for a personalized therapy. In fact, it is well recognized that genetic polymorphisms can influence clinical outcome in response to drugs [5]. In a sizable percentage of cancer patients, together with tumor regression, often occur severe and life threatening toxicities, which are of major relevance at patient and health system levels. In the post-genomic era, Pharmacogenomics (PGx) has identified genetic variants that influence both Pharmacokinetics (PK) and Pharmacodinamics (PD) [6]: PK-PGx reveal differences in patient drug metabolism and bio-availability related to gene variants involved in drug metabolism or transport, while PD-PGx analyze differences in patient response due to genomic variants producing differences in drug molecular targets/pathways [7]. In fact, it is now common notion that polymorphic variants related to Adsorption, Distribution, Metabolism and Excretion (ADME) genes significantly contribute to individual patients' drug sensitivity, resistance and toxicity. Single nucleotide polymorphisms (SNPs), genomic insertions and deletions, and genetic copy number variations (CNVs) represent the most common genetic alterations studied in PGx. SNPs are considered as common inherited variations (90%) among people, distributed throughout the genome. They represent a single nucleotide difference in the DNA sequence, which may play a functional role when occurs within a gene coding sequence or in a regulatory region. SNPs are stably inherited within haplotype blocks in *linkage disequilibrium* (LD) with a specific gene variant (LD; it is the degree to which an allele of one SNP is inherited or correlated with an allele of another SNP or a gene variant, within a population) functioning as a marker of the gene variants coheredited within the haplotype. SNPs may be therefore used in genomic analyses as tags (tagSNPs) to identify an haplotype block which may contain few or many polymorphic variants associated with a disease or drug-response phenotype (Figure 1) [8]. The frequency of a SNP is expressed as *minor allele frequency* (MAF). The identification of relevant tagSNPs [9], has allowed the evolution from a candidate-gene based research approach to the genome-wide association study (GWAS), leading to the discovery of gene variants associated to the individual risk of Adverse Drug Reactions (ADRs) and to drug efficacy because in LD with SNPs acting as tags. Recently, technologic advances have led to more cost-effective and rapid genotyping microarray platforms. Among them, Affymetrix (Santa Clara, California, USA) developed the Drug Metabolizing Enzymes and Transporters (DMET™) platform for the identification, in a single array, of all currently known polymorphisms in ADME-related enzymes, through genotyping of tagSNPs in LD [10]. The purpose of this review is to discuss the different approaches in PGx to identify predictive biomarkers on germline DNA SNPs associated to individual drug responses, with specific focus to the description of the characteristics and application of Affymetrix PGx microarray platform. We here describe the bioinformatic tools for the molecular analysis understanding and final translation into clinical practice of the information obtained by DMET™ genotyping. Moreover, we will underline advantages and weakness of statistics in PGx. Our goal is to make clear that DMET™ platform is a suitable and comprehensive PGx approach which addresses inter-individual variability in clinical response and leads to the discovery of biomarkers which, if validated, could help physician decision making for treatment personalization.

## BIOMARKERS RELATED TO TUMOR OR DRUG METABOLISM

The chance to predict and avoid ADRs, especially in the case of drugs with a narrow therapeutic index, like antitumor agents, is of major relevance in the clinical practice. Although not-inherited acquired somatic mutations in tumor tissue can influence cancer progression and drug response, other genetic alterations in transcription factor activity, gene expression, gene silencing (epigenetics), and polymorphisms are the basis of individual genetic variability. So far, a variety of novel agents have been developed for targeting specific proteins and pathways, activated by somatic mutation, on the bases of genetic alterations identified in cancer cells, like mutations involving *EGFR*, *RAS* genes, *B-RAF*, and *ALK* [11]. Somatic mutations can define disease subtypes, influence the therapeutic strategies and the clinical outcome of different tumors [12]. In almost 60% metastatic colorectal cancer (mCRC) patients, *K-RAS* and *N-RAS* are mutated and mutations are considered a predictor of poor response to anti-EGFR monoclonal antibodies (mABs), such as cetuximab or panitumumab, while patients with wild-type RAS benefit from EGFR targeted treatment [13]. Also mutations in B-RAF and *PIK3CA* (exon 20) as well as *PTEN* deletions in mCRC patients with wild-type KRAS may predict anti-EGFR resistance, but are not validated for clinical decision [14]. Inherited germline DNA polymorphisms have been identified for many proteins implicated in clinical pharmacology, and may alter bio-availability, structure, binding, and/or function, with consequent impact on drug activity and disease outcome [15, 16]. Unlike other factors influencing drug response, germline determinants generally remain stable throughout lifetime and can confer high or moderate risk for cancer susceptibility controlling which somatic mutations will undergo positive and negative selection [11, 17]. For many drugs, including anticonvulsant, anti-infective, anti-tumor, cardiovascular, opioid, proton-pump inhibitor and psychotropic drugs, a correlation has been identified between genetic variants in ADME genes and drug associations at level of cytochrome P450 (CYP) enzymes, receptors, transporters, targets and, more recently, human-leukocytes antigens (HLAs) [5]. For example, genetic polymorphism in genes coding for membrane transporters (ABCG2) and metabolism enzymes (CYP3A4, and CYP3A5, CYP1A1, CYP27B1) were correlated with the occurrence of erlotinib toxicity [18-20]. Recently, in a whole-genome sequencing of high-grade serous ovarian cancer (HGSC) tissue and germline DNA samples from 92 patients in different platinum-sensitivity status, the acquired drug resistance was associated to up-regulation of the ABCB1/MDR1 gene. The possibility to prior identify patients carriers of this drug resistant factor may allow a tailored treatment with anticancer drugs that are not a substrate of MDR1[21]. In cancer treatment, the onset of drug resistance represents an unsolved problem [22-25]. Thus, the identification of SNPs correlated to individual drug response has implemented PGx studies [26] and will offer the opportunity to select new predictive biomarkers not only for targeted therapies but also to avoid side effects associated to multi-drug regimens. Important examples of tagging SNPs in genes influencing the metabolism of antineoplastic drugs are the thiopurine methyltransferase (TPMT), involved in 6-mercaptopurine metabolism and the dihydropyrimidine dehydrogenase (DPD), involved in 5-fluorouracil (5-FU) therapy. The functional deficiency of TPMT (rs1800462 (G>C), rs1142345 (A>G) and rs1800460 (G>A)) increases the serum levels of 6-mercaptopurine with consequent serious side effects, as myelosuppression [27], while reduced DPD activity leads to prolonged 5-FU half-life and increased risk of toxicity [28]. On these bases, DMET™ Affymetrix platform allows to investigate germline polymorphisms in a panel of ADME genes, approved by the Food and Drug Administration (FDA, USA) for their involvement in drugs metabolism and elimination, in order to shed light on the complex relationships between human genetics and drug response and identify new predictive biomarkers to enhance treatment efficacy and safety.

## DIFFERENT PHARMACOGENETIC APPROACHES TO DISCOVER NEW BIOMARKERS

During the past decade the candidate gene approach has been the most widely used in the experimental design of PGx. This strategy has focused to identify genetic association between inherited variants in a single gene or a set of pathway-related genes with a clinical trait of interest, such as a drug response phenotype. Its hypothesis-driven nature implicates the knowledge of the drug pathway, metabolism or disease pathogenesis. Putative candidate genes can be drug-metabolism genes, or genes encoding drug receptors, drug transporters or proteins with important functions in pathway targeted by drugs.

Studies using this approach have led to the discovery of clinically relevant phenotype-genotype correlations, such as *CYP2D6* polymorphisms and tamoxifen activity on important clinical endpoints [29], polymorphisms in *SLCO1B1* and irinotecan pharmacokinetics and toxicity correlation [30], *DPD* variants and fluorouracil toxicity correlation [31], or *CYP27B1* and *CYP24A1* polymorphisms and non small cell lung cancer risk [32]. Although candidate gene studies can be performed with a small sample sizes to achieve the required statistical power, many associations have failed independent validation, with a high rate of false-positive, especially in cases where allelic variants are not highly penetrant [33]. Moreover, if we consider a complex disease phenotype, variations in outcome may not always be explained by one single genetic trait or one single pharmacological pathway. Thus, it is possible that multiple variants in genes involved in different processes may lead to similar phenotypic outcomes.

The development of new molecular genotyping technologies in addition to the technology advances in high-throughput analysis, have made GWAS a useful tool to simultaneously interrogate hundred to thousand of genetic variants, both SNPs and CNVs, across the entire human genome in a large number of samples. Unlike candidate gene approaches, GWAS are free of a *priori* assumption and demonstrated able not only to confirm previously-discovered PGx associations [34], but also to identify new unexpected biomarkers, associated with common disease or complex traits, for which the biological pathway was unknown [35]. Lee et al, recently, have identified by a GWAS study the correlation between a genomic variant in SLC15A2 and responsiveness to sorafenib in patients with unresectable hepatocellular carcinoma (HCC) [36]. The output produced by GWAS studies are too large to be analyzed by using common analytic packages and advanced software tools such as PLINK [37], GAINQC1, MERLIN and Mach 1.0 are required to analyze genotype-phenotype GWAS data. However, in addition to statistical association, GWAS results need further investigation to understand the mechanisms of functional effects and must be replicated in independent sample set in order to establish a causality link between a discovered gene variant and a specific trait of interest.

There are other considerations on GWAS. Common GWAS platforms are designed on LD and use a set of tagSNPs to capture all the genetic variants of the genome. However, SNPs that are in low strength of association with a tagSNP would not be detectable even if an association may indeed be found, though at lower power. In addition, GWAS can identify only common variants, with a population prevalence >5%, excluding rare alleles that, however, may have important effects on drug response. The identification of rare variants that are poorly tagged by existing genotyping platforms requires deep re-sequencing approaches for the genomic regions showing strong associations with complex traits [38]. Others important issues in GWAS are the effect size and the statistical correction for multiple testing. In discovery GWAS the expected effect sizes are unknown, and thus large study population are required to detect common variants with small effect. The sample sizes that are often used in PGx are inadequate, thus the effect sizes are often overestimated owing to the *winner's curse phenomenon.* As GWAS test large number of SNP markers, the statistical threshold used to establish a significant genetic association is typically stringent in order to avoid false positives, reducing the study's power to detect variants with small but potentially true effect.

An intermediate approach between the candidate gene studies and the GWAS is the use of pre-defined SNP list panels including thousand of genetic variants in a set of pharmacogenes. These tools combine the advantage to interrogate variants in genes selected on the basis of their known relevance in drug PK and PD with the power of simultaneous genotyping analysis, limiting the statistical correction for multiple comparisons. Alternatively, it is possible to create custom panels including only candidate genes related to specific drug-phenotype associations.

## SNPS RELATED TO DRUG METABOLISM

**Figure 2:**
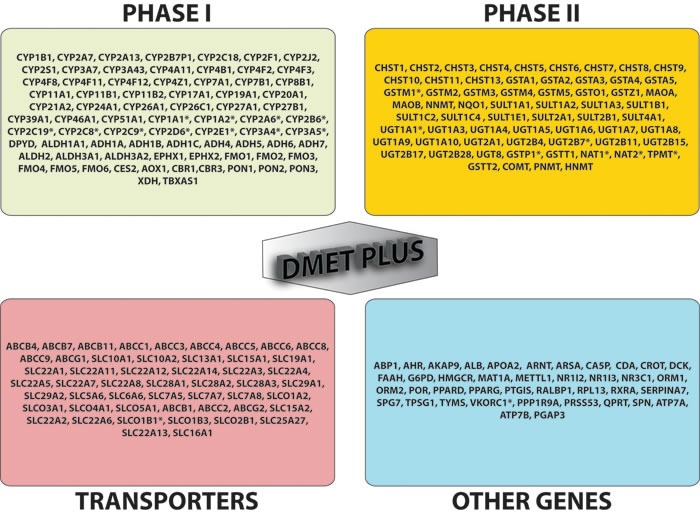
DMET gene list Genes included in DMET™ plus platform (231 total genes) are: 76 phase I enzymes, 62 phase II enzymes, 51 transporters and 41 other genes. * = translated to predicted phenotype/metabolizer status.

Many of the most relevant allelic variants involved in drug metabolism have been identified in the ADME genes encoding phase I-II enzymes and transporters. Phase I enzymes catalyze hydrolysis, reduction and oxidation reactions, and phase II enzymes catalyze conjugation reactions such as sulfation, acetylation and glucuronidation. The majority of phase I reactions are catalyzed by the CYP450 enzymes highly expressed in liver. There are 18 families of CYPs that can be further splitted into 44 subfamilies consisting of 57 total genes. However, only 3 of those families, CYP1, CYP2 and CYP3, catalyze most phase I reactions of drugs with close to 400 different unique alleles characterized to date (www.cypalleles.ki.se) [39]; over 75% of prescribed drugs are metabolized at least in part by 3 subfamilies, CYP3A, CYP2D6 and CYP2C. Otherwise, phase II drug metabolizing enzymes typically enable the biotransformation of endogenous compounds and xenobiotics and their excretion by considerably increasing the hydrophilicity of the substrate or deactivate highly reactive species as well as inactivate pharmacologically active compounds. Polymorphic variants of phase II enzymes are responsible of a reduced metabolizing capacity, which account for drug toxic effects. Also xenobiotics and pro-carcinogens are converted by phase II enzymes into highly reactive intermediates with potential activity as chemical carcinogens and mutagens by covalent binding to DNA. Specific SNPs in phase I and II enzymes are linked to phenotypes characterized by a metabolic state of “ultra” (UM), “intermediate” (IM) and “poor” (PM) metabolizers as referenced to wild-type individuals identified as “extensive” (EM) metabolizers. The PM phenotype is associated with the presence of null genotypes, IM phenotype is associated with reduced metabolism genotypes, while UM phenotype relies on gene duplications [40]. Key phase II enzymes are mostly transferases and include N-acetyltransferases 1 and 2 (NAT1 and NAT2), uridine disphosphate glucoronosyltransferase (UGTs), sulfotransferases (SULTs), glutathione S-transferases (GSTs), thiopurine S-methyltransferase (TPMT) and catechol O-methyl transferase (COMT). Also transporters are involved in the efflux and/or influx of drugs by active transport or facilitated diffusion and perform a critical role in ADME, affecting drug uptake, bioavailability, targeting, efficacy, toxicity and clearance. ATP-binding cassette (ABC) and solute-linked carrier (SLC) proteins are involved in the majority of drug and endogenous substrates transport. They act as efflux pumps and as typically influx transporters, respectively [41]. Figure 2 shows the list of 231 genes analyzed by DMET™ platform.

## GENOTYPING PLATFORMS

Platforms to analyze SNPs located in various ADME genes for pharmacological research and clinical applications have been developed [42]. Most of them are genotyping tools for the detection of polymorphisms in ADME genes of interest.

They include: i) *UGT1A1,* developed by Third Wave Technologies, Inc., which is involved in the elimination of irinotecan, ii) *CYP2C9* and *VKORC1* developed by Nanosphere, Inc., Pharagon Dx, LLC (AutoGenomics, Inc. and Luminex Corporation), which mediate warfarin metabolism and PD. The AmpliChip^®^ P450 platform, developed by Roche Diagnostics Corporation, was approved for clinical use by the FDA in 2005 to test patients for polymorphisms in the genes encoding two enzymes - CYP2D6 and CYP2C19 - that may impact on drug treatment for psychiatric illnesses [43]. The AmpliChip detects 23 SNP variants within these 2 genes, but does not identify the 39 less common SNP variants, and has already been used in the clinic and in PGx epidemiology applications and genetic research [44-46]. GE Healthcare (formerly Amersham Biosciences) produces the CodeLink™ Human P450 SNP Bioarray, which identifies 110 SNPs in nine CYP genes (Amersham Biosciences Corporation, Piscataway, NJ, USA). Recently, in 2010, Illumina, Inc. developed a platform suitable to investigate PGx variations associated with drug metabolism combining Golden Gate genotyping with VeraCode technology, that use beads probe arrays covering >95% of the PharmaADME Core list, with 184 biomarkers in 34 genes in a high throughput assay format, for many samples processing each time.

Moreover, various life science companies, including Clingenix, Inc., Epidauros Biotechnologie AG, Clinical Data, Inc. (formerly Genaissance Pharmaceuticals), Gentris Clinical Genetics, Inc. and LGC Ltd, have begun to offer genotyping services in which customers determine the genes of interest in a patient or population cohort and the company generates the SNP profiles, typically using direct gene sequencing or similar approaches. In addition, companies, including Illumina, Inc., Applied Biosystems and Sequenom, Inc., can custom design whole or targeted genome SNP platforms [42, 47] (Table 1).

**Table 1:** Genotyping platform

Manufacturer	Product	Genes investigated	Total number of variants	Registration status	Technology
Roche Molecular Diagnostics	AmpliChip CYP450 Test	CYP2C19 and CYP2D6	33 CYP2D6 alleles and 3 CYP2C19 alleles; CYP2D6 gene duplication and deletions	CE-IVD Japan-IVD US-IVD	GeneChip microarray
GE Healthcare, Amersham Biosciences	CodeLink Human P450	CYP1A1 CYP1A2 CYP3A4 CYP3A5 CYP1B1 CYP2D6 CYP2C9 CYP2C19 CYP2E1	110 SNPs and small deletions/insertion	Patent US6986992 B2	Bioarray platform, Multiplex PCR
Affymetrix, Inc	DMET™ Plus	231 ADME genes FDA approved (see Fig. 1)	1936 SNPs and 5 CNVs	For Research Use Only. Not for use in diagnostic procedures	GeneChip Microarray
Illumina	VeraCode®	ADME Core Panel	184 biomarkers in 34 genes	For Research Use Only	Beads microarray

## DMET™ PLATFORM

The number of known drug-metabolizing enzyme and transporter gene variants exceeds the capacity to assess comprehensively multiple polymorphisms by a single multiplexed assay based on current technologies such as real time-polymerase chain reaction (RT-PCR). In the last decade Affymentrix Inc. (Santa Clara, California, USA) developed the Targeted Genotyping System, which combines molecular inversion probe (MIP). This technology is an oligonucleotide-based method that can be used to analyze several thousand SNPs in a single assay developed by Hardenbol et al [48], and extensively used for the International HapMap project, that offers several advantages for multiplex genotyping [49]. It is based on ‘padlock probes’, which are oligonucleotide probes (connected by a linkage segment) that recognize two complementary genomic sequences [49]Based on the MIP technology, Affymetrix developed a multiplex within the PharmaADME consortium. The consortium ranked over 9000 SNPs and many complex mutations within these genes (i.e., triallelic markers, small in/del mutations, gene conversion and/or whole deletion alleles) according to clinical research utility. Currently, PharmaADME genes represent 95% (45/47) of the phase I enzymes, 93% (74/80) of the phase II enzymes, 98% (51/52) of the transporters, and 52% (24/46) of ‘other genes’ on the DMET™ array. The DMET™ panel was modified to include 37 additional genes (i.e., 231 genes total), mostly comprising genes that regulate intracellular processes that facilitate ADME (i.e., scaffolding proteins, nuclear receptors, serum binding proteins etc). The genes presented in the DMET™ platform were selected by their ‘VIP’ status on PharmGKB. Recently, Affymetrix has added additional content relevant to drug ADME, and a tool to identify haplotypes among 779 polymorphisms in a core set of 61 genes identified by the PharmaADME consortium of high-relevance in drug metabolism. Moreover, the platform identifies additional haplotypes that were not previously observed in populations, explored by the HapMap project. The DMET™ platform has been designed to capture several markers, including copy-number variations, insertions/deletions, biallelic and triallelic SNPs, but until now its use is intended for research only because it doesn't hold FDA approval for *in vitro* diagnostic devices (IVD) marked assay.

### Analytical procedure

**Figure 3:**
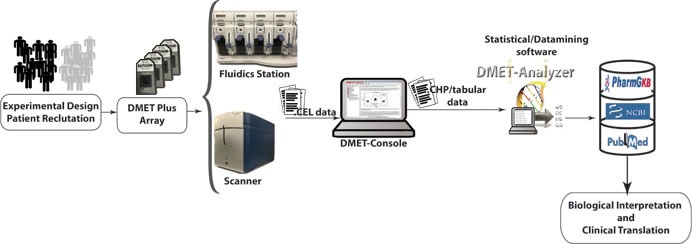
DMET data analysis workflow

The DMET™ assay uses 1μg of genomic DNA samples diluted in Tris-EDTA buffer, extracted from peripheral blood or saliva [50]. The protocol start with an initial PCR amplification step to amplify 32 loci that either has a pseudo gene or do not generate sufficient signal using the routine “Targeted Genotyping” protocol. These pre-amplified products are then combined with genomic DNA then incubated with a multiplex anneal cocktail PCR included in the “Targeted Human DMET™” assay probe panel. The remaining steps are carried out according to Affymetrix protocol, then arrays are scanned with 4-color detection using the Affymetrix GeneChip Scanner. Raw signal values are background subtracted and normalized, and genotypes are reported using the Affymetrix DMET^®^ Console software as single-sample genotyping by comparing each individual marker's data to the specific, predefined cluster boundaries (Figure 3). For a given marker in a particular sample, the collection of summary values is reduced to only two values, one for each allele for simple bi-allelic variants. Genotypes are determined for each SNP site and reported as homozygous wild-type, heterozygous, homozygous variant or ‘no call’. The DMET™ Plus Assay Panel has been evaluated across a minimum of 1200 individuals from multiple populations including 597 DNA samples from Caucasian, African, and Asian populations from the International HapMap Consortium to assess accuracy, imprecision, and dynamic range. Genotyping accuracy varies across the core set probes. Specifically, the reproducibility of genotyping results for the core set probes rates of approximately 98% for within- and between-day runs, globally about 98%, with the majority of failures resulting from lack of a call, defined as no-call (NC) or possible rare allele (PRA). This imprecision of the assay is acceptable for this complex assay. Moreover, the use of the PRA designation is helpful in this regard because, despite introduce a high false-positive rate, is useful as a screening test to be confirmed as a definitive genotype call by alternative methods. This could be a conservative approach, since all discrepancies with direct sequencing data are counted as errors. Moreover, the most frequent assay failure is the lack of a genotype call defined as NC/PRA, rather than a miscalled genotype, that is a critical point to make a distinction for clinics. In fact, an absence of data is less problematic than assignment of an incorrect genotype to a patient.

Another weak point of genotyping done by this assay is that allele quantification is not possible in the current format: thus large-scale deletions or duplications like CYP2D6*5 [51] or *1XN [52] cannot be readily detected. Of course, homozygous deletions can be inferred if low signals across multiple probes for a gene are detected. As well as, particularize small tandem repeats like TA repeats in UGT1A1*28 [53] is difficult with the current format of the assay.

It must be taken into account that the multiplex nature of this assay maintains low the cost considering that it does not scale up by increasing the number of tested variants, allowing large-scale genotyping at a acceptable cost. Therefore, this approach may be useful to aid the comprehension of complex multigenic interactions that impact PK beyond the more simple monogenic models. In fact, it uses a single-microarray assay that allows for the comprehensive genetic analysis of genes involved in drug metabolism, transport, and excretion. Considering that a microarray-based approach permits that rare variants can be included in the assay with no appreciable increase in its complexity or cost, this assay results strongly powerful. The Affymetrix DMET™ platform includes an extensive list of genes involved in drug disposition, and may become an important tool for future PGx research. Nevertheless, certain limitations and caveats warrant attention. First and foremost, the DMET™ platform has not undergone, to our knowledge, evaluation to FDA as IVD, and cannot, therefore be used to inform clinical decisions. Thus DMET™ cannot, for example, be used to test patients prospectively to determine warfarin or irinotecan dose requirements, or for decision making on antiplatelet therapy. Whether Affymetrix intends to undergo FDA review for this device is not publicly known at the time of the current report. The implementation of this tool in the early stage of drug development may be of major relevance for the identification of patients at risk for ADRs providing a method to investigate better tailoring of drug regimens for individual patients. The platform could conceivably be applied to the study of other complex genetic interactions as the correlation between a PK/PD biomarker and the tumor phenotype. In fact, the understanding of the underlying relationship between drug exposure, biomarker and drug effect is crucial for the identification of clinically relevant outcome predictors and to assess their optimal evaluation timing. We believe that this tool will be critical for understanding the complex multigene interactions underlying drug metabolism and the integration with PK/PD tools can allow to analyze simultaneously both longitudinal biomarker and survival data, as in the current vision of precision medicine.

### PGx analysis and interpretation: DMET^®^ Console

**Figure 4:**
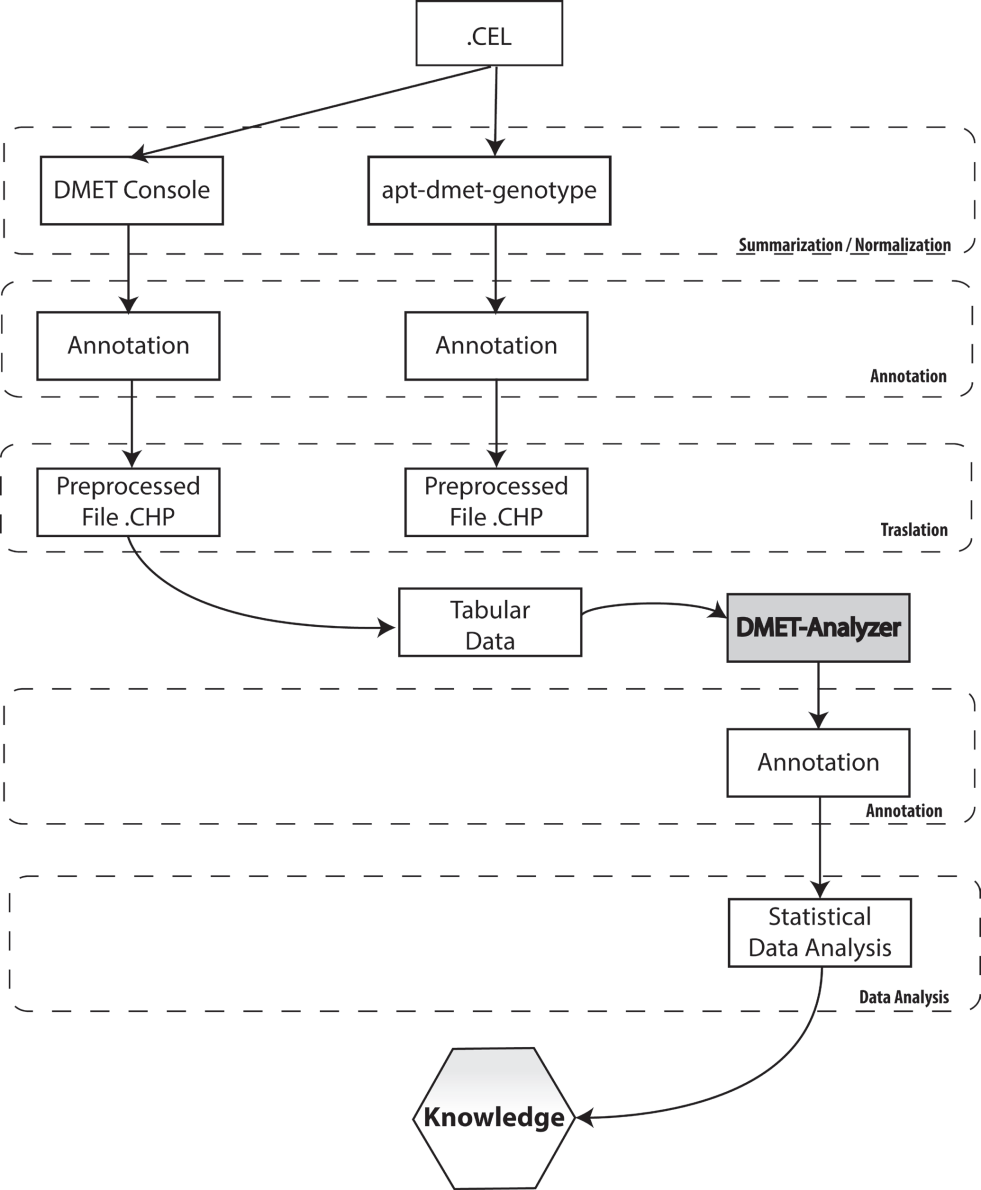
Statistical analysis and interpretation The picture describes necessary steps to convert intensity value in actionable knowledge. Each column represents the flow of information when using respectively DMET^®^ Console, apt-DMET-genotype and DMET-Analyzer.

In order to extract biological relevant information embodied in the raw data produced using microarray, and stored as CEL files, it is necessary to translate CEL files in a format suitable to conduct statistical or data mining analysis. A typical workflow for analyzing microarray data involves four steps: i) preprocessing, that comprises background correction, summarization and normalization; ii) annotation and translation; iii) statistical/data mining analysis; and iv) biological interpretation. Three different tools: DMET^®^ Console, apt-DMET-genotype, DMET Analyzer (see below) can be used to convert intensity value in actionable knowledge (Figure 4). Background correction adjusts probe intensities ensuring that background corrected signal is always positive. Summarization aims to recognize the position of different genes in raw images, associating different regions of pixels to the unique gene that generated them. Normalization corrects the variation of gene expression in the same array due to experimental bias, making results from different microarray experiments comparable. The summarization/normalization of CEL files can be done only using DMET^®^ Console and apt-DMET-genotype, because DMET-Analyzer is not designed to treat directly CEL file format. Files produced into the summarization/normalization step can be annotated only using DMET^®^ Console and apt-DMET-genotype. Using DMET^®^ Console, it is possible get tabular data, by means a step known as translation, where CHP file and ARR sample files are merged together, to translate intensity value using standardized nomenclature. The annotation process associates to each gene a set of functional information, for example the biological function related with the gene. Translation converts the genotype calls (reported in CHP files) of an important subset of marker, to functional allele calls using standardized nomenclature wherever possible. In terms of biological research it is very important to identify the small set of variation into the genes called SNP, comparing two experimental conditions (e.g. healthy cell *vs* cancer cell, wild type *vs* mutant). After the pre-process layer, tabular data provided by DMET^®^ Console can be automatically analyzed by DMET-Analyzer. There are several univariate statistical methods used later to pinpoint mutated genes that may contribute to the development of a certain disease from normalized microarray data, including T-tests, Chi-Square, Fisher's Test, and Bayesian models. DMET-Analyzer by Fisher's exact Test extract knowledge hidden into the data in a format easily readable from the user. Data mining methodologies are very useful as well as statistical analysis, helping to discover interesting unknown relationships hidden into the data then converted in a understandable way to the user. Furthermore, to perform analysis in an efficient way, tabular data need further preprocessing. In the preprocess layer, DMET-Analyzer arranges data in a format compatible for the statistical assay. In the annotation layer, preprocessed data are annotated with information provided by Affymetrix or using information coming from external databases i.e. dbSNP or Pharmacogenomics Knowledge Base (PharmGKB.) Finally, biological interpretation allows for each analyzed SNP, to obtain additional information stored in the Pharma-GKB [54] (Figure 4).

## PHARMACOGENOMIC STUDIES USING THE DMET™ PLATFORM

Since the first DMET™ platform became available in the late 2007, several researchers have used the platform to conduct correlative PGx studies. The pioneeristic study was conducted by Caldwell et al. that investigated whether the consequences of genetic variants in addition to the previously identified effects of *CYP2C9* and *VKORC1* may explain inter-patient variability in response to the anticoagulant drug warfarin (Coumadin) [55]. Warfarin is the oral anticoagulant, approved by the US FDA, commonly used in atrial fibrillation and thromboembolic disease. Even it has been introduced more than 50 years ago, the treatment can still be complicated by wide inter-individual variations in the dose required to achieve the biological effect. Polymorphisms in the cytochrome P450 (CYP) 2C9 and in vitamin K 2,3 epoxide reductase complex 1 (VKORC1) genes were associated with the inter-individual variability in the dose-anticoagulant effect of warfarin [56-61]. Caldwell et al. using the DMET panel genotyped an initial discovery cohort of patients (n = 497) from the Marshfield Clinic and identified a single variant in the *CYP4F2* gene (rs2108622) that correlated with warfarin dose requirements. Cohorts of patients from 2 additional institutions were used to confirm these results using different genotyping methodologies. Aside from 100% concordance rate with the DMET panel results, the authors evidenced again that the rs2108622 SNP correlated with warfarin dose requirements [55].

Thereafter, the DMET™ platform was used to evaluate pharmacological variation in prostate cancer patients randomized to phase II clinical study with docetaxel and thalidomide *versus* docetaxel alone. Both anticancer agents showed inter-individual pharmacological variation and toxicity profile [62]. Past PGx studies explored factors mediating docetaxel PK and thalidomide toxicity have no led to consistent results due to the large variability observed. By the use of a more comprehensive analysis of genetic polymorphisms in multiple drug enzymes and transporters, improved the understanding of the PK of docetaxel and thalidomide. DMET genotyping, identified statistically significant correlations between SNP variants and drug response or toxicity highlighting a role of non-CYP450 enzymes in the pharmacology of docetaxel and thalidomide [62]. By DMET™ platform, Uchiyama T et al. identified one SNP in *CYP39A1* gene (rs7761731) significantly associated with grade 4 neutropenia in Japanese patients with gynecological cancers that may be a useful biomarker for predicting the risk of docetaxel-induced neutropenia [63].

Mega et al. used the DMET™ platform to explore the PK and PD of clopidogrel, an anti-platelet agent used to treat patients with coronary disease [64]. Clopidogrel is a prodrug that requires activation by CYP enzymes, and has demonstrated significant inter-individual PD variability in inhibiting platelet aggregation [65]. Among patients who had experienced myocardial infarction and had been treated with clopidogrel in the TRITON-TIMI clinical trial (ClinicalTrials.gov identifier: NCT00357968), the authors identified individuals carrier of *CYP2C19*allele that produces a reduced-functionhad significantly lower levels of the active metabolite of clopidogrel, diminished platelet inhibition, and a higher rate of major adverse cardiovascular events, including stent thrombosis [64]. In a successive study, among acute coronary syndrome patients treated with clopidogrel, Mega et al. identified that *ABCB1* C3435T genotype was significantly associated with risk for the primary endpoint of cardiovascular death, myocardial infarction or stroke. The authors described that *ABCB1* C3435T and *CYP2C19* genotypes were significant, independent predictors of the primary endpoint, and that the 47% of the population, who were either *CYP2C19* reduced-function allele carriers, *ABCB1* 3435 TT homozygotes, or both were at significantly increased risk of cardiovascular death, myocardial infarction, or stroke. Moreover, in healthy subjects, the presence of *ABCB1* C3435T TT homozygotes had a reduction in platelet aggregation with clopidogrel respect to CT/CC individuals disclosing less platelet inhibition and were at significantly increased risk of recurrent ischemic events in the setting of clopidogrel treatment. Considering both*ABCB1*and*CYP2C19* genetic polymorphisms, nearly half of the population are carries of genotype and then associated with an increased risk for major adverse cardiovascular events while on standard doses of clopidogrel [66]. These results lead FDA's approval of drug label for clopidogrel that contains a boxed warning, stating that clopidogrel has diminished effectiveness among CYP2C19 poor metabolizers. It advises that tests are available to identify a patient's *CYP2C19* genotype, which may be of help for determining therapeutic use, and that alternative treatment strategies should be considered in patients identified as CYP2C19 poor metabolizers [67]. The Clinical Pharmacogenetics Implementation Consortium has released anti-platelet therapy recommendations based on *CYP2C19* genotype for patients affected by acute coronary syndrome and undergoing percutaneous coronary interventions, such as the placement of a stent. Given the reduced efficacy reported for both CYP2C19 intermediate and poor metabolizers, recommends using an alternative antiplatelet agent. Recently, Erlige et al. compared results obtained with the Nanosphere Verigene^®^ System, a novel genetic test capable of analyzing 11 *CYP2C19* variants within 3 hours, to the established and validated DMET genotyping method for identifying extensive and reduced metabolizers of clopidogrel. Based on genotyping, statement from the Clinical Pharmacogenetics Implementation Consortium, patients with stable coronary artery disease on clopidogrel 75 mg daily are defined as extensive metabolizers (*1/*1, *1/*17, *17/*17), reduced metabolizers (*1/*2, *1/*8, *2/*2, *2/*3), or of indeterminate metabolizer status (*2/*17). The Nanosphere Verigene^®^ System identified 11 *CYP2C19* alleles in less than 3 hours with a high degree of accuracy when compared to conventional method, and was further validated against PK and PD phenotypes [68].

The role of UDP-glucuronosyltransferase (UGT)1A1 (UGT1A1*28) in determining the toxicity induced by irinotecan is well known [69, 70]. Recently, by DMET ™ platform, Di Martino et al. identified 3 SNPs mapping in *ABCG1*, *ABCC5* and *OATP1B1*/*SLCO1B1* transporter genes associated with gastrointestinal toxicity grade ≥3, induced by irinotecan in metastatic colorectal cancer in a case control study. The SNP rs562 in *ABCC5*, the rs425215 in *ABCG1* and the rs2306283 in *OATP1B1*/*SLCO1B1* polymorphisms expand the available knowledge of irinogenomics [71]. Moreover, DMET polymorphisms have been associated with toxicity to a new nanopharmaceutical formulation of camptothecin, specifically designed for slowly release of the drug in tumors over an extended time [72]. Specifically, the authors performed genotyping of a small number of patients experiencing toxicity (15) and compared the allele frequencies with Affymetrix HapMap population (713). The study appears however unbalanced and the heterogeneous population did not allow sound comparisons. In a different case-control study, Di Martino et al. [73] identified a peroxisome proliferator-activated receptor gamma (PPARG) polymorphism (rs1152003) associated with zoledronic acid-related osteonecrosis of the jaw in multiple myeloma (MM) patients. This finding is of potential relevance in the treatment of MM-related bone disease. Osteolytic bone disease represents in fact a major hallmark of a paradigmatic evolving disease that represents a challenging field for novel therapeutics development [74-82]. In this context, bisphosphonates, which have deep biological effects within the bone microenvironment, remain the cornerstone of skeletal events management in this disease [83-88]. Identifying patients with increased susceptibility to osteonecrosis of the jaw might significantly impact in supporting strategies for this important malignancy. More recently, the same authors, with similar approach identified 7 SNPs in 6 genes (*CYP27B1*, *MAT1A1*, *CHST1*, *CYP4B1*, *ADH6*, and *SLC22A1*) associated with the occurrence of skin rash in advanced non-small cell lung cancer treated with erlotinib [20]. In this study, the toxicity-associated gene set underwent to *Ingenuity Pathway Analysis*^®^ highlighting the involvement of 1,25-dihydroxyvitamin D3 biosynthesis, S-adenosyl-L-methionine biosynthesis, and methionine degradation I (to homocysteine) canonical pathways in skin rush development. Although exploratory, this study suggests new mechanism mediated by vitamin D3 and inflammation at skin level, which appears highly relevant to shed new light in the erlotinib-related skin toxicity.

5-FU is commonly used in the treatment of solid tumors. However, 5-FU activity and toxicity can be influenced by dihydropyrimidine dehydrogenase (DPYD) and thymidylate synthase (TYMS) gene polymorphisms. In colorectal cancer samples, Rumiato et al. found polymorphisms with the strongest association with 5-FU-induced gastrointestinal toxicity, such as the rs9787901 in *CHST1* and rs1799735 in *GSTM3* genes that have not been previously related to 5-FU PK and PD [89].

More recently, different studies using an updated DMET™ platform led to the identification of new polymorphisms in various ADME genes, previously not investigated. For example, the contribution of *SLCO1B3* and *UGT1A* polymorphisms to the PK of telmisartan, commonly used to treat hypertension, was investigated at microdose (MD,100 μg) and at therapeutic dose (TD, 80 mg). Authors observed strong LD between UGT1A1*6 and UGT1A3*4a, and between UGT1A1*28 and UGT1A3*2a in terms of effect on the PK of telmisartan, while no obvious effect was observed for SLCO1B3 polymorphisms. Specifically, following MD or TD injection, the mean area under the curve 0-24 (±standard deviation) of telmisartan was significantly higher in individuals with the UGT1A3*2a and *4a variants compared to those in individuals with UGT1A3*1/*1, and quantitatively correlated with population PK analysis. These findings led the authors to the conclusion that UGT1A3 haplotypes significantly influence PK of telmisartan, results that are potentially important for pharmacological and toxicological evaluation [90].^.^

Paclitaxel is a cytotoxic drug frequently used in the treatment of a variety of cancers associated with different severe adverse events. The development of paclitaxel-induced peripheral neuropathy has been described from several groups to be primarily influenced by drug exposure and patient polymorphisms in *CYP2C8* gene [91]. Specifically the CYP2C8*3 polymorphism has been associated to peripheral neuropathy risk due to decreased metabolism and elimination, which leads to increased toxicity and efficacy mainly in African-Americans. By DMET genotyping analysis, Hertz et al. described breast cancer patients with higher paclitaxel-related neuropathy risk in the CYP2C8 low-metabolizer group, that carried the CYP2C8*2, *3, or *4 variant. However, the influences of the *2 and *4 SNPs were not independently significant in this study. In addition one intronic SNP, the rs492338 in *ABCG1*, showed strong association with neuropathy in the Caucasian cohort (p = 0.0008), but not in the non-Caucasian validation group (p = 0.54). Even if the PGx heterogeneity is present in the cohort of breast cancer patients, it does not directly influence the risk of neuropathy beyond the contribution of CYP2C8*3 [92]. Moreover, based on the DMET™ platform, by the application of the nonlinear mixed-effect modeling software ( NONMEM ,version 7, Icon Development Solutions) for placlitaxel PK evaluation, it has been developed a genetic prediction model including 14 SNPs with high sensitivity to identify patients with low paclitaxel clearance but which is not able to explain differences in paclitaxel clearance [93]. A similar 10-SNP model was not able to reach statistical significance in order to predict paclitaxel-induced neutropenia [94]. Therapeutic activity of standard platinum-based neoadjuvant therapy in esophageal cancer patients is variable and unpredictable. At present, no reliable response predictors could discriminate between responder and non-responder patients. By DMET™ array platform Rumiato et al, identified 16 SNPs significantly associated with good or poor response while no association was found for 4 variants mapping in DNA repair machinery. The predictive power of *ABCC2*, *ABCC3*, *CYP2A6*, *PPARG*, and *SLC7A8* gene variants was demonstrated and a predictive model for sentitivity to platinum-based neo-adjuvant chemotherapy was built combining clinical variables and the genetic signature [95]. The corrrelation of genetic variation analyzed by DMET™ Plus platform and response to treatment in acute myeloid leukemia (AML) has been investigated in CD33-positive AML patients enrolled in a phase III multicenter clinical trial combining Gemtuzumab-Ozogamicin (GO) with Fludarabine-Cytarabine-Idarubicin (FLAI) regimen, [96]. In this study authors showed significant differences in allele frequencies of two *ADH1A* variants between patients with therapeutic benefit and not responders. Two substitutions on *CYP2E1* and one on *SLCO1B1* were found to differentially influence hepatic toxicity, and two nucleotide changes on *SULTB1* and *SLC22A12* genes correlated with GO treatment benefit. All these variants are associated with differential response and toxicity in AML patients treated with a combination of GO-FLAI regimen [96]. A genetic variant in *SLCO1B1* (rs2291075; c.597C>T), encoding the transporter OATP1B1, has been recently associated with event free and overall survival in children with de novo AML [97]. The results of this study lead the authors to argue that the lack of SLCO1B1 expression in leukemic blasts might be due to inherited rather than somatic effect. In addition, the authors demonstrated by *in vitro* functional studies that 4 AML-directed drugs (cytarabine, daunorubicin, etoposide, and mitoxantrone) are substrates for OATP1B1, underlining its important role in the PK of multiple anti-AML drugs and suggesting that inherited variability in host transporter function influences the efficacy of therapy [97].

Thompson et al. investigated the impact of obesity, body composition, and genetic polymorphisms on the PK of daunorubicin in children with cancer. Performing PGx profiling by DMET™ platform the authors identified association of *FMO3* and *GSTP1* haplotypes with daunorubicin PK, suggesting a potential role in the efficacy and toxicity of the drug [98].

The mechanisms of small intestine damage induced by aspirin is not well understood but is increasingly recognized as risk factor for bleeding. Shiotani et al by DMET analysis identified an association of GG genotype in *CYP2D6* gene (rs28360521) with small bowel bleeding and SNPs in *CYP4F11* and *CYP2D6* were proposed as risk markers for aspirin toxicity [99]. Different studies have previously shown the association of the *SLCO1B1* 521TT genotype and the SLCO1B1*1b haplotype with the risk of aspirin induced peptic ulcer [100]. More recently, they performed PGx profile by DMET™ platform in a series of patients taking 100 mg of aspirin. They found that the frequencies of the SLCO1B1*1b haplotype and *CHST2* 2082 T allele were higher in peptic ulcer patients [101].

DMET™ platform was also used in an exploratory PGx approach to investigate the inter-individual PK variability in busulfan, a drug used in conditioning regimens before stem cell transplantation. In this study SNPs in *GSTA5* gene (rs4715354 and rs7746993) were significantly associated with busulfan clearance confirming a role of the glutathione-S-transferases and its relation to outcome in adult hematopoietic stem cell recipients [102].

All together, these studies indicate DMET™ microarray platform as highly efficient approach to discover new genetic determinants influencing chemotherapy-induced toxicity as well as to identify different metabolizing phenotypes. Moreover, the high concordance of DMET genotyping results with orthogonal technologies like real-time PCR and direct sequencing is of major relevance. These findings indicate that DMET™ platform is an excellent tool to incorporate PGx tests into prospective clinical research. We summarize the results obtained by DMET™ platform in the Table 2.

**Table 2:** Pharmacogenomics studies by Affymetrix DMET™ Plus

Drug	Disease	Phenotype	Sample size	Gene	SNP(s)	Reference
Warfarin	Cardiovascular disease	Clinical response	497	*CYP4F2*	rs2108622	[50]
Docetaxel and/or Thalidomide Docetaxel	Prostate cancer Gynecological cancer	Clinical response Toxicity Neutropenia	47 42	*PPAR-γ* *SULT1C2* *CHST3* *SPG7 CYP2D6 NAT2 ABCC6* *ATP7* *CYP4B1 SLC10A2* *CYP39A1*	rs2016520^a^, rs1883322^a^ rs3734254^a^, rs7769719^a^ rs6922548 rs1402467 rs4148943, rs4148947, rs12418, rs730720 rs2292954, rs12960 rs72549353 rs1799931 rs2238472 rs2227291 rs4646487 rs2301159 rs7761731	[57] [58]
Clopidogrel	Cardiovascular disease	Clinical response Clinical outcome	162 2932	*CYP2C19* *ABCB1*	rs4244285 rs1045642	[59] [61]
Irinotecan	Colorectal cancer	Gastrointestinal toxicity	26	*ABCC5* *ABCG1* *SCLO1B1*	rs562 rs425215 rs2306283	[66]
Zoledronic acid	Multiple Myeloma	Osteonecrosis of the jaw	19	*PPARG ABP1* *CHST11* *CROT*	rs1152003 rs10983, rs4725373, rs1049793 rs2463437, rs903247, rs2468110 rs2097937	[68]
Erlotinib	Advanced Non Small Lung cancer	Skin rush	34	*CYP27B1* *MAT1A* *CHST11* *ADH6* *CYP4B1*	rs8176345 rs9285726 rs903247, rs2468110 rs6830685 rs2297809	[84]
5-Fluorouracil	Colorectal cancer	Toxicity	24	*CHST1* *GSTM3*	rs9787901 rs1799735	[85]
Telmisartan	Hypertension	Pharmacokinetics	33	*UGT1A1* *UTG1A3*	rs4148323, rs8175347 rs3806596, rs45625338	[86]
Paclitaxel	Breast cancer Solid tumors	Peripheral neuropathy Clearance	209 412 270	*CYP2C8* *CYP2C8* *ABCG1* *SLC22A11* *GSTZ1* *SLC28A2* *VKORC1* *PGAP3* *CDA* *EPHX1* *CYP20A1* *SLC6A6* *CRIP3* *GSTA4* *AKAP9* *CYP51A1* *CYP2D7P1*	rs10509681 rs10509681 rs492338 rs1783811 rs7975 rs1060896 rs9923231 rs2952151 rs1048977 rs1051740 rs1048013 rs2341970 rs2242416 rs13197674 rs7785971 rs7797834 rs28360521	[87] [88] [89]
Fludarabine-Cytarabine-Idarubicin	Acute Myeloid Leukemia	Clinical response Toxicity	94	*ADH1A* *SULT2B1* *SLC22A12* *CYP2E1* *SLCO1B1*	rs6811453, rs1826909 rs2302948 rs11231825 rs2070673, rs2515641 rs4149056	[92]
Ara-C-daunorobucin-etoposide-mitoxantrone	Acute Myeloid Leukemia	Overall survival	164	*SLCO1B1*	rs2291075	[93]
Daunorubicin	Hematological cancers	Clearance	107	*FMO3 GSTP1*	rs2266782 rs1695	[94]
Aspirin	Cerebrovascular disease	Small bowel bleeding	25	*CYP2D6* *CYP4F11*	rs28360521 rs1060463	[95]
Aspirin	Cardiovascular disease	Peptic ulcer Ulcer bleeding	593	*SLCO1B1* *CHST2*	rs4149056 rs6664	[97]
Busulfan	Hematological cancers	Clearance	65	*GSTA5*	rs4715354, rs7746993	[98]

a Results are from analyses restricted to docetaxel and thalidomide trial arm

## DMET™ *VERSUS* GWAS

In a PGx study design, sample size is crucial in conditioning strength and statistical validation of biomarker discovery. As previously discussed, while GWAS has been the cornerstone of gene variant identification, several pitfalls have been identified in the last years if GWAS might be used as the unique approach for gene association PGx studies.

It has to be underlined that GWAS studies are generally aimed to the discovery of hidden associations among allelic variants and phenotypic effect in a large population, while DMET studies are usually tailored to smaller populations. GWAS studies allow the identification of a haplotype by a tagSNP but do not allow to fully assess the contributions of a gene relevant to drugs due to a non-uniform coverage of all the chromosomes or chromosomal regions. DMET™ platform allows the haplotype association and in addition, is able to identify the single SNP diplotype validated for its involvement in drug metabolism and rarer variations increasing the power to identify association in PGx studies [103]. A further consideration regards the quality of DMET data and how they are reliable. As noted by Fernandez et al. [104], DMET genotypes are accurate and results are high reliable. Conversely, due to the high dimensionality of genome-wide arrays, GWAS studies have difficult application in the clinical context, while tailored arrays for PGx purposes, such as DMET™, may achieve better results in clinical context as reported by Gamazon et al [103].

Therefore, about the different goals and data analysis approaches by DMET™ and GWAS, some points must be made clear: (i) the specific aim of the analysis, (ii) the data dimensionality, and (iii) the statistical (or data mining) models. The second point is preponderant, from a computer science point of view, since it has direct relations with the choice of the analytical model for the study aim. For the first point, the goal of study design should be considered and consequently the sample size suitable: GWAS studies investigate associations among genetic variants and phenotypes on broad aspects, while DMET studies are tailored to the investigation of PGx. About the data dimensionality, it should be noted that DMET experiments consider 1936 allelic variants while a typical GWAS study may consider up to 906,600 variants (e.g. in Affymetrix SNP 6.0 array). Consequently, the analysis of GWAS data poses relevant challenges of data dimension. A single file containing data of a SNP 6.0 array has a typical dimension of some Giga Bytes, while a file containing DMET data has a size of some Kilo Bytes. This feature requires the introduction of *ad hoc* solutions (e.g. high performance data management) for the analysis of GWAS data. Considering the statistical models, it should be noted that the analysis of GWAS data is a broader field that involves both statistical and data-mining approaches [104], while DMET data analysis mainly relies on the use of Fisher's Test or association rules [105, 106]. We here summarize some main approaches (Table 3) and readers may refer to Fernandez et al. for a broader coverage of this relevant topic [104]. Association studies made by GWAS are usually performed using logistic regression for dichotomous studies or Fisher's exact tests for simpler studies. For continuous traits, linear regression has been used for GWAS. Data mining has also been used to perform discriminant analysis among cases and controls using decision trees. Finally, statistical models of analysis present some common point and differ for dimension and aim of the analysis. It is important to note that for a GWAS study it is mandatory to evaluate the statistical power of the study before performing the experiments. In fact, the high number of variables may cause the poor statistical significance of the found associations [107]. There exist some statistical tools that evaluate the power of the studies and are able to predict the needed number of patients (or samples) to be enrolled in a study. The minimum number of samples is, in general, very high, limiting the use of GWAS in clinical context or in PGx context, since often meta analysis should be performed in order to have a significant number of samples.

**Table 3:** Comparison of DMET™ and GWAS Data

	DMET™	GWAS
Study design	Studies are usually tailored to the study of small populations.	Studies aim to discover hidden associations among allelic variants and phenotypic effect in a large population.
Dimension of data	Around Kilo-bytes	Up to 1Giga-byte
Data analysis	Data analysis mainly relies on the use of Fisher's exact Test or association rules.	Data analysis is a broader field that involves both statistical and data-mining approaches.

## BIOMARKER VALIDATION PROCESS

In a living organism, a biomarker is a characteristic hallmark precisely measured and objectively validated that describes a normal or abnormal biological state, pathogenic processes or predict pharmacologic responses to a therapeutic treatment [108]. The process for biomarker validation, after the discovery in basic studies implies multiple processes including the validation in an independent clinically relevant cohort of patients.

In the discovery studies, a set of patients is enrolled to identify a biomarker according to the study design and the primary endpoint (training set). Biomarker validation is usually carried out by testing the same set of samples by both the assay used in the discovery study and the clinical deployment platform, in order to assess the robustness and reproducibility of the measurements. According to study design, for determining the reliability and quality of biomarkers and in the aim to reduce the sources of bias the guidelines to be followed are REMARK for prognostic studies [109], STROBE for observational studies [110] and STARD for diagnostic studies [111]. The independent patient validation cohort enrollment is a crucial step to demonstrate that the biomarker are generalizable outside the learning cohort. Following validation the next step is candidate biomarker qualification achieved by the development and optimization of an assay platform for its measurement including sensitivity, specificity and reproducibility. This step is subjected to two types of validation: analytical and clinical validation. Analytical validity of an assay is the ability to detect accurately and reliably the selected biomarker in the laboratory and in samples representative of the clinical population under investigation while the clinical validation is the correlation of the candidate biomarker to a clinical endpoint [112]. The analytical validation is performed by testing the assay used in the initial discovery and the clinical deployment platform on the same set of samples to verify robustness and reproducibility of the measurements. According to FDA in this phase are identified as ‘probable’ valid biomarker process that don't have the necessary scientific control, and ‘known’ valid biomarker process that achieved widespread agreement [113]. The final step will be the clinical implementation that must be compliant with different regulatory processes in the European Community (CE) and United States (US) and proceed from regulatory approval to incorporation in clinical practice guidelines [112] as FDA-cleared or*CE*-*IVD*markedclinical diagnostic tests. Commonly to other predictive biomarker assays their validation is intended for a specific use (specific tissue type, specific patient population, and specific collection method).

## FUTURE APPLICATIONS

**Figure 5:**
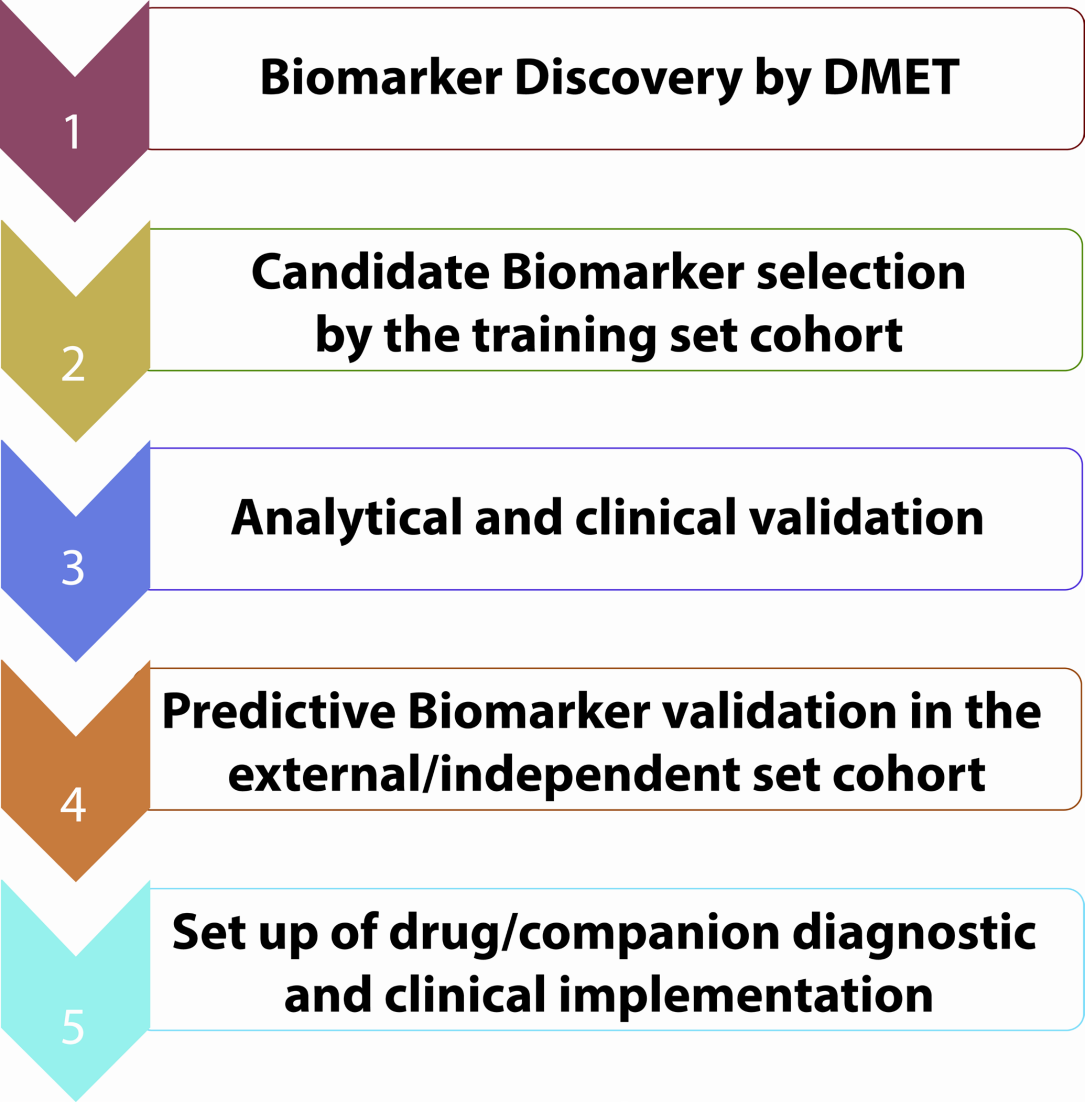
Biomarkers validation workflow

**Figure 6:**
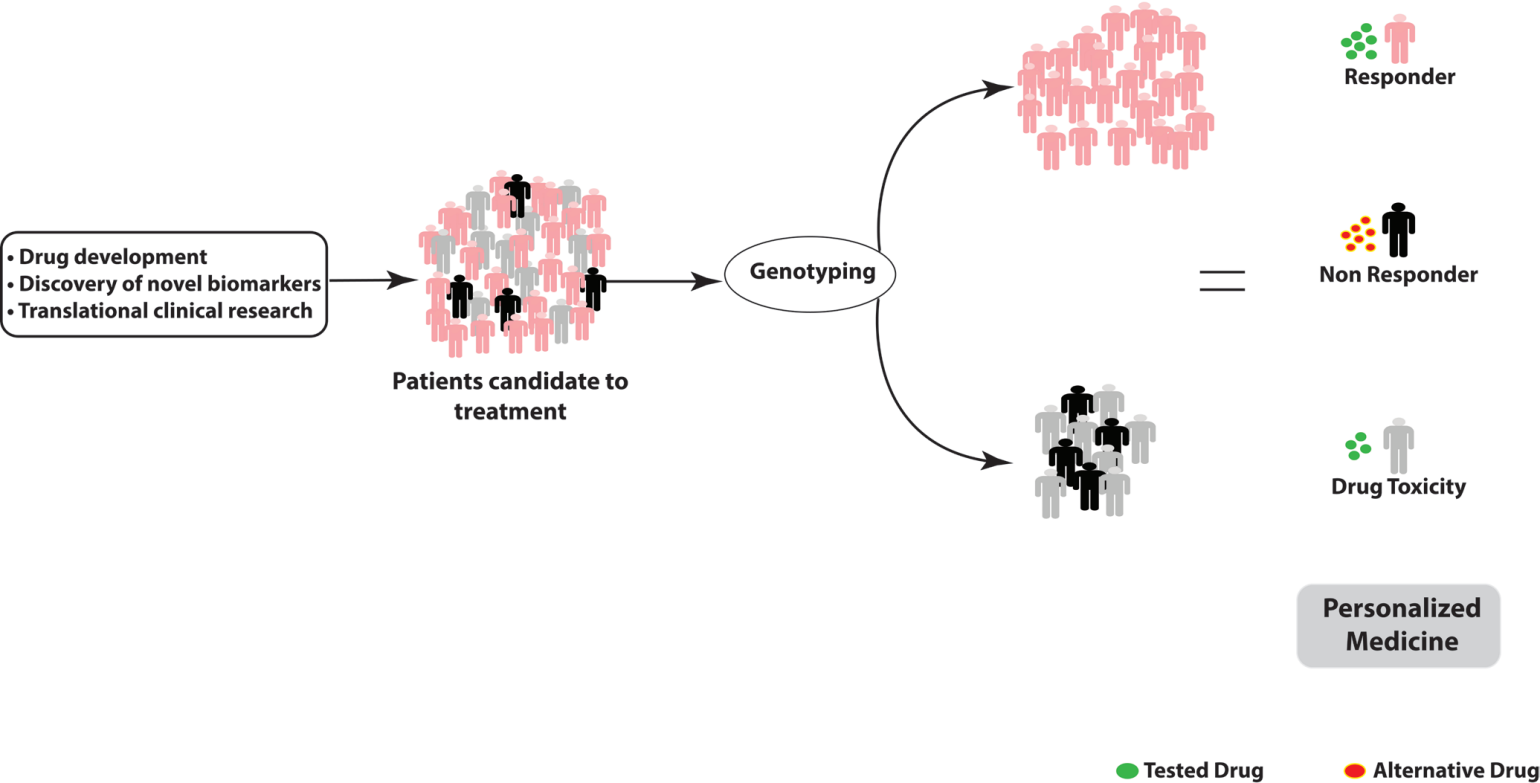
Genotyping platform for personalized therapy: genetic variants in pharmacodynamics and pharmacokinetics related genes determine inter-individual variability and therapeutical outcome Patients predicted as non responder should undergo treatment with alternative drugs; patients predicted at risk of drug toxicity should undergo dose reduction.

Genetically determined variations in ADME genes can affect inter-individual heterogeneity in drug response. The availability in clinical practice of predictive biomarkers for response to commonly used drugs could help physicians in daily practice and improve patient care with relevant benefits to health systems. At present, the chance to empower clinical practice by the application of PGx findings is not immediately feasible in the real world practice, and strong efforts are still required to translate scientific discoveries into therapeutic options. So far, several SNPs are potential predictive PK/PD biomarkers. Some of them are already included in the drug sheet as for the UGT1A1*28 in the case of irinotecan. In our vision, this innovative approach should be included in personalized medicine algorithm for cancer management. A similar approach in CRC might include mutation analysis of *NRAS*, *KRAS*, *BRAF* and immune microenvironment typing, which might allow treatment selection on the basis of an integrated view [114, 115]. In our opinion, the future goal for personalized cancer therapy will be in fact the knowledge of patient's specific genetic background to pre-select patients not fit for a given treatment, at risk of severe and life-threatening toxicities. In this scenario, DMET™ platform may allow selection of candidate biomarkers to translate, after validation, on custom platform for different diseases requiring specific treatments in order to set up the companion diagnostic for clinical practice and to increase the safety and the efficacy of the drug (Figure 5). Until now, clinically predictive biomarkers are included only in the last phases of drug validation process: the hope is that diagnostic tools and drug development might integrate their paths for a co-development to allow an improvement of their clinical utility in terms of patients health outcomes. In this way, it will be possible to withheld treatment of patients genetically at risk for ADRs. The routine application of DMET driven genotyping should be included in prospective clinical trials (Figure 6). The identification of novel molecular targeted compounds, should include PK/PD prevision by DMET analysis, in order to produce a development path in the era of precision medicine. In this view, algorithms will be required to integrate molecular data with drug mechanisms and/or disease knowledge [116, 117].

An additional emerging point is the impact of environmental factors such as lifestyle, diet and co-medications on drugs PK/PD, and the profiling of CYP450 enzymes involved in metabolic activation of several pro-carcinogens [118]. PGx investigations on genome-disease, genome-drug interactions and drug disease interactions will allow to evaluate their potential role as biomarkers related to cancer risk and susceptibility in clinical studies designed to find novel ways to prevent cancer. Analysis of DMET data would allow the study of the molecular mechanisms underlying interaction between polymorphic variants in ADME genes and xenobiotics metabolism, improving PGx information on cancer susceptibility. The identification of the disease molecular basis and the understanding that germline DNA mutations can influence drugs response [119-121] and disease outcome have given a great impulse to PGx studies, and DMET™ PGx approach has the potential to improve the identification phase of new biomarkers for personalized medicine. The integration of DMET-driven biomarkers with the novel genetic information provided by high-throughput “omics” technologies might represent an innovative approach to open new scenarios towards precision medicine in oncology and for the design of new clinical investigations.
